# Supplementary material for: Association of small, dense LDL-cholesterol concentration and lipoprotein particle characteristics with coronary heart disease: A systematic review and meta-analysis
Source: PLoS One. 2020 Nov 9;15(11):e0241993. doi: 10.1371/journal.pone.0241993 (PMC7652325; doi:10.1371/journal.pone.0241993)
Supplement: S1 File — (DOCX) [file pone.0241993.s001.docx]

# **SUPPLEMENTARY APPENDIX**

**Supplementary Methods**

**Standardization of Exposures**

Studies either reported an association of sdLDL and CHD or sdLDL-C and CHD. sdLDL/sdLDL-C was measured at the individual level using a variety of assays summarized in Table 1. Studies reported sdLDL either as a binary variable indicating presence or absence of sdLDL or a continuous variable of sdLDL concentration. The majority of sdLDL-C studies reported ORs by groups of sdLDL-C concentration (mg/dL). Because the associations were most frequently reported as quartiles (with the bottom quartile as reference), the measures of association in the few studies reporting other comparisons were first transformed to similarly represent the top versus the bottom quartile comparison (Equation 1). The measures of association for the studies that used a continuous exposure of sdLDL were transformed to reflect a binary exposure (Equation 2).

| ${lnOR}_{std}={CF}_{1}*{lnOR}_{measured}$ | (Eq. 1) |
| --- | --- |
| ${lnOR}_{std}={CF}_{2}*{lnOR}_{measured}/\sigma$ | (Eq. 2) |
| CF_1_ = conversion factor of 2.54/difference in means of given intervals  CF_2_ = conversion factor of 1.6/difference in means of given intervals | |

This transformation was performed under the assumption that the exposure variable was normally distributed (21), and a log-linear association existed between exposure and outcome, as previously described (23).

**Justification of Pooling Different Measures of Association**

Two different measures of association were reported in the reviewed studies: odds ratios and hazard ratios, which were calculated using logistic and Cox regression respectively. Hazard ratio and relative risk are broadly equivalent if the risks are relatively constant over time (24), and under the rare disease assumption, the relative risk and odds ratio are equivalent. Thus, the two can be reasonably equated to each other, and risk was used in this essay. In one systematic review, CHD was found to have an age-adjusted prevalence of 8% in the 60-65 age group (the median age in most of the included studies) (25), which is reasonably rare. For studies that reported multiple measures of association, the maximally adjusted ones were used in the primary analysis.

**S1 Figure.** Forest plot of association between (A) sdLDL, (B) sdLDL-C and CHD subgrouped by degree of covariate adjustment


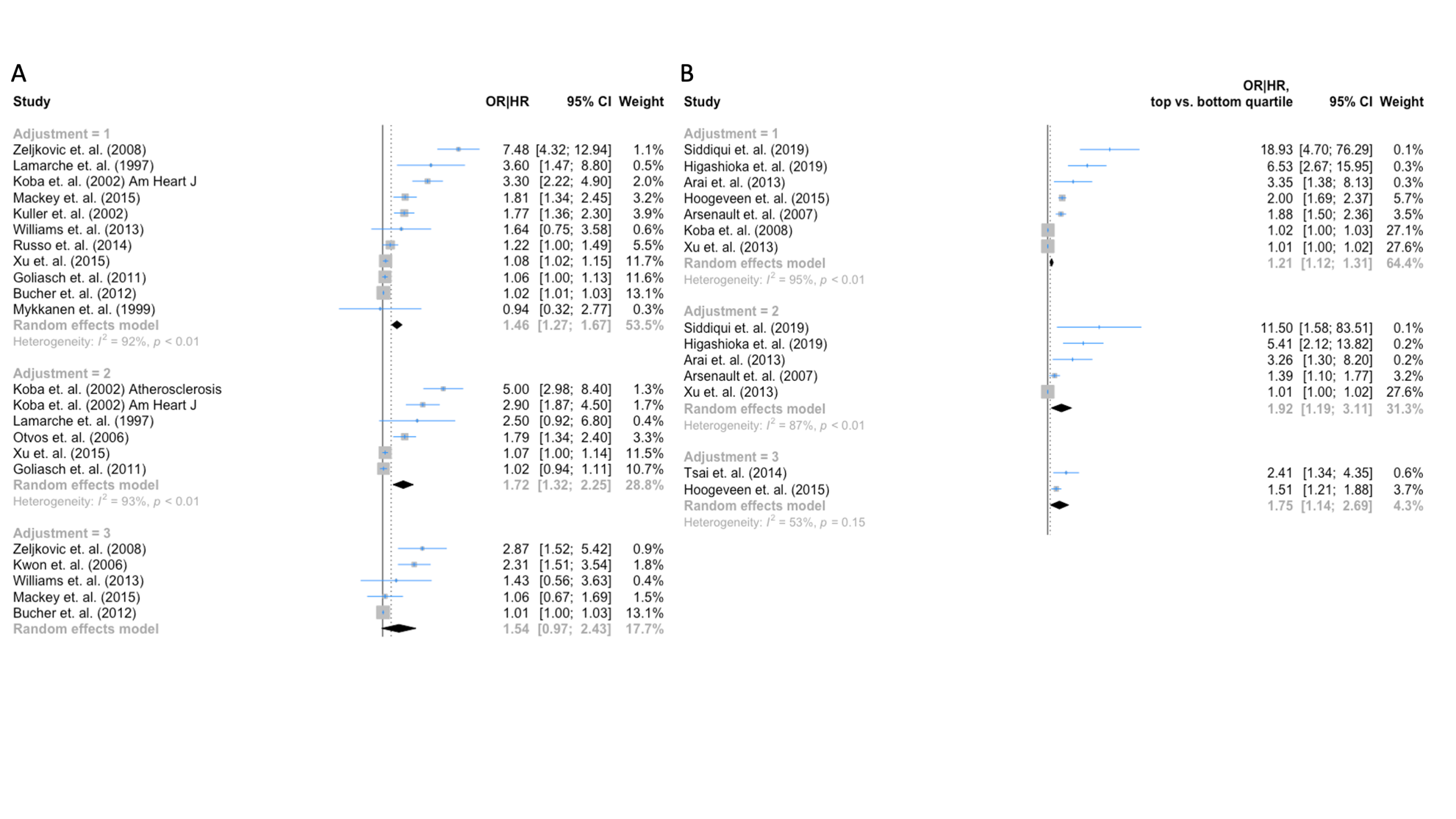


‘1’= unadjusted or adjusted for only sex and age; ‘2’= adjusted for demographics and lifestyle risk factors; ‘3’= adjusted for demographics, lifestyle risk factors and lipid subfractions. Maximally adjusted hazard ratios from each study were used. Note that there were no sdLDL studies that only adjusted for lipid subfraction

**S2 Figure.** Funnel plot of (A) sdLDL studies and (B) sdLDL-C studies contributing to pooled effect on CHD

**
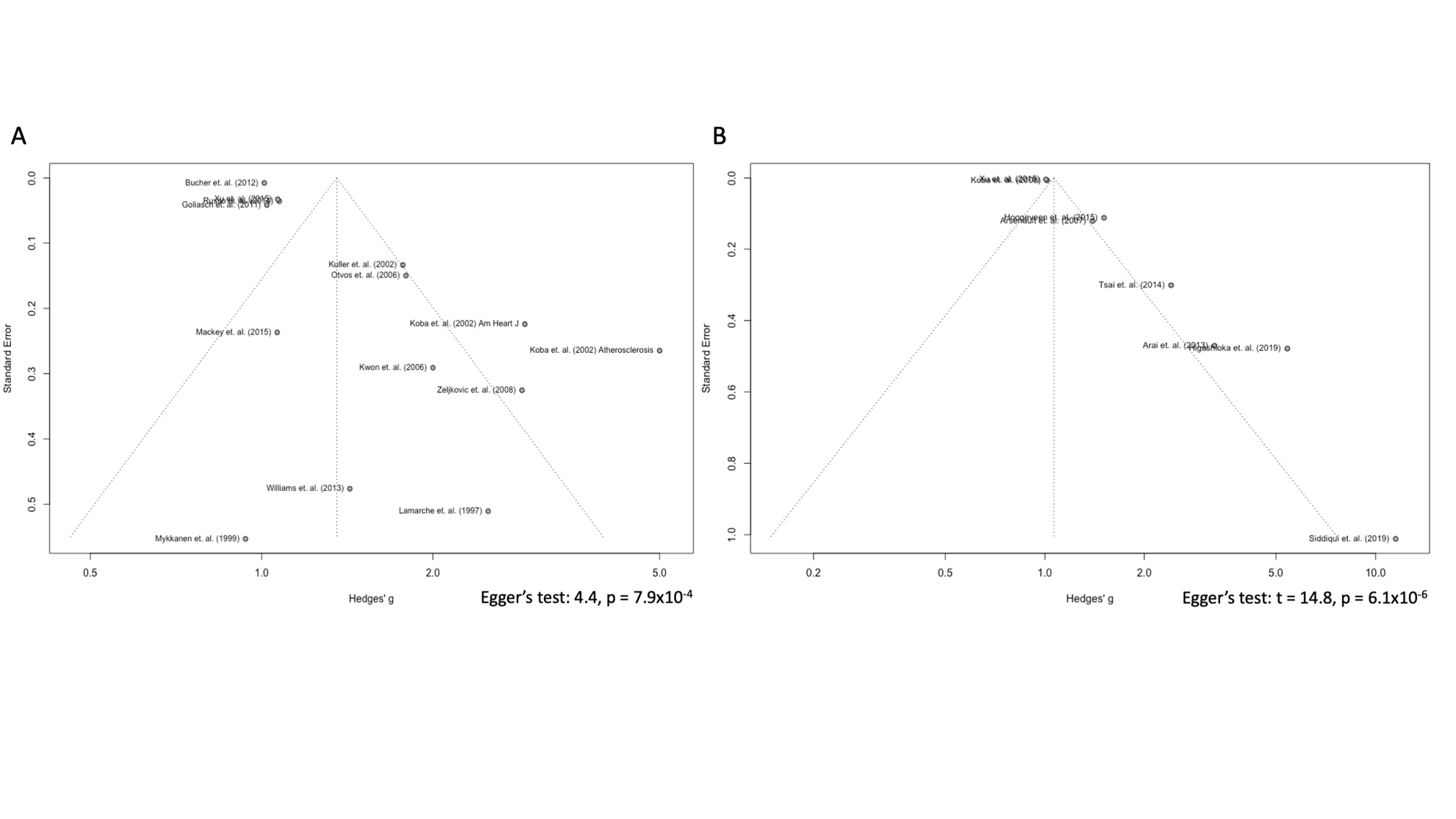
**

**S1 Table.** Search strategy

| **PubMed** |
| --- |
| (("Small dense low-density lipoprotein cholesterol"[TIAB] OR "sdLDL-C"[TIAB] OR "small dense LDL"[TIAB] OR “small LDL”[TIAB]) AND ("Coronary Heart Disease"[TIAB] OR "Coronary Artery Disease"[TIAB] OR “Ischemic Heart Disease”[TIAB] OR “Ischaemic Heart Disease”[TIAB]) AND (“HR”[TIAB] OR “Hazard Ratio”[TIAB] OR “OR”[TIAB] OR “Odds Ratio”[TIAB])) |
| **MEDLINE** |
| (("Small dense low-density lipoprotein cholesterol,ti.ab." OR "sdLDL-C,ti.ab." OR "small dense LDL.ti,ab." OR “small LDL.ti,ab.” OR “dense LDL.ti,ab.” OR “dense LDL cholesterol”.ti,ab.) AND ("Coronary Heart Disease.ti,ab." OR "Coronary Artery Disease.ti,ab." OR “Ischemic Heart Disease.ti,ab.” OR “Ischaemic Heart Disease.ti,ab.”) AND (“HR.ti,ab.” OR “Hazard Ratio.ti,ab.” OR “OR.ti,ab.” OR “Odds Ratio.ti,ab.”)) |
| **EMBASE** |
| (("Small dense low-density lipoprotein cholesterol,ti.ab." OR "sdLDL-C,ti.ab." OR "small dense LDL.ti,ab." OR “small LDL.ti,ab.” OR “dense LDL.ti,ab.” OR “dense LDL cholesterol”.ti,ab.) AND ("Coronary Heart Disease.ti,ab." OR "Coronary Artery Disease.ti,ab." OR “Ischemic Heart Disease.ti,ab.” OR “Ischaemic Heart Disease.ti,ab.”) AND (“HR.ti,ab.” OR “Hazard Ratio.ti,ab.” OR “OR.ti,ab.” OR “Odds Ratio.ti,ab.”)) |
| **Web of Science** |
| TS=("Small dense low-density lipoprotein cholesterol" OR "sdLDL-C" OR "small dense LDL" OR “small LDL” OR “dense LDL” OR “dense LDL cholesterol”) AND TS=("Coronary Heart Disease" OR "Coronary Artery Disease" OR “Ischemic Heart Disease” OR “Ischaemic Heart Disease”) AND TS=(“HR” OR “Hazard Ratio” OR “OR” OR “Odds Ratio”) |

**S2 Table.** Assessment of Risk of Bias using the CLARITY tool

| **1st Author/Year of Publication** | **Study Design** | **expass** | **outcomeabs** | **match** | **prog** | **outass** | **follow** | **develop** | **casesel** | **controlsel** | **Total** | **Risk Category** |
| --- | --- | --- | --- | --- | --- | --- | --- | --- | --- | --- | --- | --- |
| Arai et. al. (2013) | PC | 1 | 1 | 2 | 1 | 1 | 1 | 0 | 0 | 0 | 7 | Low |
| Arsenault et. al. (2007) | NCC | 1 | 0 | 2 | 0 | 1 | 1 | 2 | 1 | 1 | 9 | Low |
| Bucher et. al. (2012) | NCC | 1 | 0 | 1 | 0 | 1 | 1 | 1 | 1 | 1 | 7 | Low |
| Goliasch et. al. (2011) | CC | 1 | 0 | 2 | 0 | 1 | 0 | 1 | 1 | 1 | 7 | Low |
| Higashioka et. al. (2019) | PC | 1 | 1 | 2 | 1 | 1 | 1 | 0 | 0 | 0 | 7 | Low |
| Hoogeveen et. al. (2015) | PC | 1 | 2 | 2 | 1 | 1 | 1 | 0 | 0 | 0 | 8 | Low |
| Koba et. al. (2002) Atherosclerosis | CC | 1 | 0 | 4 | 0 | 1 | 0 | 1 | 1 | 1 | 9 | Low |
| Koba et. al. (2002) Am Heart J | CC | 1 | 0 | 4 | 0 | 1 | 0 | 1 | 1 | 1 | 9 | Low |
| Koba et. al. (2008) | CC | 1 | 0 | 4 | 0 | 1 | 0 | 1 | 1 | 1 | 9 | Low |
| Kuller et. al. (2002) | CC | 1 | 0 | 4 | 0 | 1 | 0 | 1 | 1 | 1 | 9 | Low |
| Kwon et. al. (2006) | CC | 1 | 0 | 2 | 0 | 2 | 0 | 1 | 1 | 2 | 9 | Low |
| Lamarche et. al. (1997) | NCC | 1 | 0 | 1 | 0 | 1 | 1 | 1 | 1 | 1 | 7 | Low |
| Mackey et. al. (2015) | NCC | 1 | 0 | 0 | 0 | 1 | 1 | 1 | 1 | 1 | 6 | Low |
| Mykkanen et. al. (1999) | NCC | 1 | 0 | 4 | 0 | 1 | 3 | 1 | 1 | 2 | 13 | Moderate |
| Otvos et. al. (2006) | RCT | 1 | 0 | 2 | 0 | 1 | 1 | 1 | 1 | 1 | 8 | Low |
| Russo et. al. (2014) | CC | 1 | 0 | 4 | 0 | 1 | 0 | 3 | 3 | 3 | 15 | Moderate |
| Siddiqui et. al. (2019) | PC | 1 | 2 | 2 | 1 | 1 | 1 | 0 | 0 | 0 | 8 | Low |
| Tsai et. al. (2014) | PC | 1 | 1 | 2 | 1 | 1 | 1 | 0 | 0 | 0 | 7 | Low |
| Williams et. al. (2013) | RCT | 1 | 0 | 0 | 0 | 1 | 1 | 0 | 0 | 0 | 3 | Low |
| Xu et. al. (2015) | CC | 1 | 0 | 1 | 1 | 1 | 1 | 1 | 1 | 1 | 8 | Low |
| Zeljkovic et. al. (2008) | CC | 1 | 0 | 4 | 0 | 2 | 0 | 1 | 1 | 1 | 10 | Low |

**S3 Table.** Original reported measures of association and standardization method

| **Study** | **Original Measure of Association** | **Exposure** | **Quantification of Exposure** | **Standardization Method** |
| --- | --- | --- | --- | --- |
| Arai et. al. (2013) | 3.26 | sdLDL-C | Quartiles (4 v1) | N/A |
| Arsenault et. al. (2007) | 1.33 | sdLDL-C | Tertiles (3 v 1) | $\frac{2.54*ln(1.33)}{2.18}$ |
| Bucher et. al. (2012) | 1.04 | sdLDL | Per 1 mg/dL | $\frac{1.6*ln(1.04)}{7.9/1.35}$ |
| Goliasch et. al. (2011) | 1.06 | sdLDL | Per 1 IQR of 6 mg/dL | $\frac{1.6*ln(1.06)}{6/1.35}$ |
| Higashioka et. al. (2019) | 5.41 | sdLDL-C | Quartiles (4 v1) | N/A |
| Hoogeveen et. al. (2015) | 1.51 | sdLDL-C | Quartiles (4 v1) | N/A |
| Koba et. al. (2002) Atherosclerosis | 5 | sdLDL | Binary (1=Presence, 0=Absence) | N/A |
| Koba et. al. (2002) Am Heart J | 2.9 | sdLDL | Binary (1=Presence, 0=Absence) | N/A |
| Koba et. al. (2008) | 1.027 | sdLDL-C | Per 1 mg/dL | $\frac{2.54*ln(1.027)}{4.21}$ |
| Kuller et. al. (2002) | 1.77 | sdLDL | Binary (1=Presence, 0=Absence) | N/A |
| Kwon et. al. (2006) | 2.312 | sdLDL | Binary (1=Presence, 0=Absence) | N/A |
| Lamarche et. al. (1997) | 2.5 | sdLDL | Binary (1=Presence, 0=Absence) | N/A |
| Mackey et. al. (2015) | 1.45 | sdLDL | Per 1 SD | $1.6*ln(1.45)$ |
| Mykkanen et. al. (1999) | 0.96 | sdLDL | Per 1 SD | $1.6*ln(0.96)$ |
| Otvos et. al. (2006) | 1.44 | sdLDL | Per 1 SD | $1.6*ln(1.44)$ |
| Russo et. al. (2014) | 1.224 | sdLDL | Per 1 mg/dL | $\frac{1.6*ln(1.224)}{4.53}$ |
| Siddiqui et. al. (2019) | 4.657 | sdLDL-C | Binary, 25 mg/dL | $\frac{2.54*ln(1.33)}{1.6}$ |
| Williams et. al. (2013) | 1.36 | sdLDL | Per 1 SD | $1.6*ln(1.36)$ |
| Tsai et. al. (2014) | 2.41 | sdLDL-C | Quartiles (4 v1) | N/A |
| Xu et. al. (2015) | 1.079 | sdLDL | Binary (1=Presence, 0=Absence) | N/A |
| Xu et. al. (2015) | 1.03 | sdLDL-C | Per 1 mg/dL | $\frac{2.54*ln(1.03)}{9.83}$ |
| Zeljkovic et. al. (2008) | 2.867 | sdLDL | Binary (1=Presence, 0=Absence) | N/A |

Standardized measures of association provided in Figure 3.

**S4 Table.** Detailed description of characteristics of reviewed studies

| **Study** | **Setting** | **Population studied** | **Outcome definition** | **Adjustment** |
| --- | --- | --- | --- | --- |
| **Prospective Cohort** |  |  |  |  |
| Arai et. al. (2013) | Suita, Japan | Random sample selected from Suita of men and women aged 30-79 | first-ever acute MI, sudden cardiac death within 24 h after the onset of acute illness, or CAD followed by coronary artery bypass surgery or angioplasty | age, sex, BMI, smoking (never, ex, current), drinking (never, ex, current), blood pressure, diabetes, and lipid-lowering drugs |
| Higashioka et. al. (2019) | Hisayama, Japan | Full community survey of Hisayama residents over the age of 40 | first-ever fatal and nonfatal MI, silent MI, sudden cardiac death within 1 h after the onset of acute illness, coronary angioplasty, and bypass grafting | age, sex, blood pressure, antihypertensives, HbA1c, glucose-lowering agents, BMI, eGFR, electrocardiogram abnormalities, current smoking, current drinking, regular exercise |
| Hoogeveen et. al. (2015) | 4 US communities | Men and women aged 45-64 sampled from the general population | MI, fatal CHD, or cardiac procedure | age, sex, race, smoking, BMI, hypertension, HDL-C, log(trigylcerides), lipid-lowering medication, diabetes, diabetes medications, log(hs-CRP) |
| Russo et. al. (2014) | Messina, Italy | Type 2 diabetic postmenopausal women | nonfatal MI, CHD death | creatinine clearance, age |
| Siddiqui et. al. (2019) | Hospital in Virgina, USA | Liver transplant recipients recruited at Virginia Commonwealth University between January 2012 and January 2014 | chronic ischemic heart disease, MI, coronary-artery bypass, coronary angioplasty | Liver disease, ethnicity, sex, hypertension, obesity, statin use |
| Tsai et. al. (2014) | 6 communities in the US | Men and women aged 45-84 without clinical evidence of CHD were sampled | angina, MI, need for revascularization, cardiac death | sex, blood pressure, hypertension medication use, HDL-C, log(triglycerides), age, race, lipid lowering, diabetes |
| **Nested Case Control** |  |  |  |  |
| Arsenault et. al. (2007) | Norfolk, UK | Random sample selected from Norfolk of men and women aged 45-79 | ICD codes 410-414 for death due to CHD | age, smoking, diabetes, BMI, blood pressure, LDL-C, date of visit or FRS which takes into account age, sex, total cholesterol, HDL-C, SBP, DBP, smoking, diabetes |
| Bucher et. al. (2012) | 7 centers in Switzerland | HIV-infected individuals in Switzerland | definitive MI, possible MI or unstable angina, coronary artery bypass grafting, coronary angioplasty or stenting, or fatal coronary event based on D:A:D Study | age, sex, smoking status, total cholesterol, trigylcerides, HDL cholesterol, systolic blood pressure, central obesity, diabetes, family history of CHD, IV drug use, years of antiretroviral therapy, years on abacavir, HIV viral load, nadir CD4 cell count |
| Lamarche et. al. (1997) | 6 suburbs of Quebec metropolitan area | Men aged 35-64 were randomly sampled from the general population using provincial electoral lists | typical effort angina, coronary insufficiency, nonfatal MI, and coronary death | smoking, BMI, alcohol, diabetes, medication use, family history of CAD, blood pressure |
| Mackey et. al. (2015) | 40 centers in the US | Obese postmenopausal women aged 50-79 at 40 centers in the US between 1993 and 1998 who chose not to or were ineligible to participate in WHI hormone or diet clinical trials | MI, angina, revascularization, congestive heart failure, stroke, peripheral vascular disease | age, ethnicity, BMI, waist circumference, smoking, diabetes, hypertension, physical activity, lipid-lowering drugs, hormone therapy, LDL |
| Mykkanen et. al. (1999) | Kuopio, Finland | White men and women aged 65-74 randomly sampled from Kuopio | Not provided | sex, diabetes |
| **Case Control** |  |  |  |  |
| Goliasch et. al. (2011) | 2 centers in Vienna | Cases: Patients with acute myocardial infarction aged at most 40; Controls: Recruited from other hospital departments free from previous AMI | premature MI | age, sex, BMI, hypertension, diabetes, smoking, family history of CAD |
| Koba et. al. (2002) Atherosclerosis | Hospital in Showa, Japan | Cases: Men with and without diabetes with CAD; Controls: Men with and without diabetes without CAD | acute MI, previous MI, stable angina pectoris, unstable angina pectoris, vasospastic angina | age, sex, diabetes |
| Koba et. al. (2002) Am Heart J | Hospital in Showa, Japan | Cases: Men and women with CHD who underwent coronary arteriography at thehospital from May 1999 to October 2000 | acute MI, previous MI stable angina pectoris, unstable angina pectoris, vasospastic angina | age, sex, diabetes |
| Koba et. al. (2008) | Hospital in Showa, Japan | Cases: Men and women with CHD who underwent coronary arteriography at the hospital from April 2004 to September 2005 | MI, angina pectoris | LDL-C, HDL-C, apoA-1, Apo B, non-HDL-C, HbA1c, lipid-lowering drugs |
| Kuller et. al. (2002) | 4 communiities in the US | Recruited from a defined sample of Medicare files over the age of 65 | angina, stroke, MI, transient cerebral ischemia | None |
| Kwon et. al. (2006) | Hospital in Yonsei University | Cases: Men and women showing CAD by coronary angiogram between October 2003 and June 2004 | stenosis of one or more coronary artery branch with 50% of the diameter or more luminal narrowing as seen by coronary angiography | age, obesity, smoking, hypertension, diabetes, low HDL-C, high LDL-C |
| Xu et. al. (2014) | FuWai hospital | Patients referred for elective coronary angiography due to angina-like chest pain and/or positive treadmill exercise test or coronary CT angiography | One or more diseased epicardial vessels with a diameter of more than 2 mm that had at least a 50% diameter stenosis | age, sex, BMI, hypertension, diabetes, current smoking, family history |
| Zeljkovic et. al. (2008) | Hospital in Serbia | Men and women aged 28-74 all hospitalized at the Institute of Cardiovascular Diseases, Clinical Centre of Serbia | Not provided | age, triglycerides, HDL, apoA-I |
| **Randomized Controlled Trial** | | | | |
| Otvos et. al. (2006) | 20 Veterans Affairs medical centers in the US | Men younger than 74 years with an established diagnosis of CHD | CHD death, nonfatal MI | age, medication use, hypertension, smoking, BMI, diabetes |
| Williams et. al. (2013) | United States and Canada | Patients were recruited on the basis of having had clinical coronary disease and low HDL cholesterol (<35 mg/dl for men and <40 mg/dl for women) | Death from coronary causes, nonfatal myocardial infarction, stroke, or revascularization for worsening ischemia | age, sex, BMI, current smoking status, lipid subfractions |

**Abbreviations:** CHD = coronary heart disease; CAD = coronary artery disease; MI = myocardial infarction; BMI = body mass index; FRS = Framingham Risk Score; LDL-C = low density lipoprotein cholesterol; HDL-C = high density lipoprotein cholesterol; SBP = systolic blood pressure; DBP = diastolic blood pressure; eGFR = estimated glomerular filtration rate; hs-CRP = high -sensitivity C-reactive protein, apoA-1 = apolipoprotein A-1; HbA1c = hemoglobin A1c

**S5 Table.** Detailed summary of sdLDL assays used in reviewed studies

| **Study** | **Serum/plasma** | **Storage Temperature (°C)** | **Assay** | **Details** | **Validity** |
| --- | --- | --- | --- | --- | --- |
| Arai et. al. (2013) (sdLDL-C) | Plasma | -80 | Hitachi 7180 automated analyzer provided by Denka Seiken | Automated homogenous assay developed by Ito et al. that dissociates non-LDL lipoproteins and protects sd-LDL particles. Cholesterol concentration is then measured by a standard assay. | This method has good agreement with the ultracentrifugation methods, a standard procedure for the measurement of sdLDL. There was some evidence of compositional changes of sdLDL particles (d < 1.044 kg/L) using this method. |
| Arsenault et. al. (2007) (sdLDL-C) | Serum | -80 | Non-denaturing 2-16% polyacrylamide gradient gel electrophoresis from Bio-Rad |  | Limitations include expensive equipment, complicated laboratory techniques, and long assay times. |
| Bucher et. al. (2012) (sdLDL) | Plasma | -70 | Ultracentrifugation, Synchron CX5 assay |  | Limitations include expensive equipment, complicated laboratory techniques, and long assay times. |
| Goliasch et. al. (2011) (sdLDL) | Plasma | -80 | Quantimetrix LDL and HDL Lipoprint® system | Polyacrylamide gel electrophoresis-based method to measure cholesterol in lipoprotein fractions | Limitations include expensive equipment, complicated laboratory techniques, and long assay times. |
| Higashioka et. al. (2019) (sdLDL-C) | Serum | Unspecified | Hitachi 7180 automated analyzer provided by Denka Seiken | Automated homogenous assay developed by Ito et al. that dissociates non-LDL lipoproteins and protects sdLDL particles. Cholesterol concentration is then measured by a standard assay. | This method has good agreement with the ultracentrifugation methods, a standard procedure for the measurement of sdLDL. There was some evidence of compositional changes of sdLDL particles (d < 1.044 kg/L) using this method. |
| Hoogeveen et. al. (2015) (sdLDL-C) | Plasma | Unspecified | Hitachi 917 automated analyzer provided by Denka Seiken (LDL-EX) | Automated homogenous assay that dissociates non-LDL lipoproteins. Cholesterol concentration is then measured by a standard assay. | This method has good agreement with the ultracentrifugation methods, a standard procedure for the measurement of sdLDL. There was some evidence of compositional changes of sdLDL particles (d < 1.044 kg/L) using this method. |
| Koba et. al. (2002) Atherosclerosis (sdLDL) | Plasma | Unspecified | 2-16% polyacrylamide gel |  | Limitations include expensive equipment, complicated laboratory techniques, and long assay times. |
| Koba et. al. (2002) Am Heart J (sdLDL) | Serum | 4 | 2-16% polyacrylamide gel |  | Limitations include expensive equipment, complicated laboratory techniques, and long assay times. |
| Koba et. al. (2008) (sdLDL-C) | Serum | 4 | Hitachi 917 automated analyzer provided by Denka Seiken (LDL-EX) | Automated homogenous assay that dissociates non-LDL lipoproteins. Cholesterol concentration is then measured by a standard assay. | This method has good agreement with the ultracentrifugation methods, a standard procedure for the measurement of sdLDL. |
| Kuller et. al. (2002) (sdLDL) | Plasma | -80 | NMR |  | NMR has the advantage of being able to determine the size and number of LDL particles, but it is very costly. |
| Kwon et. al. (2006) (sdLDL) | Serum | Unspecified | Quantimetrix LDL and HDL Lipoprint® system | Polyacrylamide gel electrophoresis-based method to measure cholesterol in lipoprotein fractions | Limitations include expensive equipment, complicated laboratory techniques, and long assay times. |
| Lamarche et. al. (1997) (sdLDL) | Plasma | -86 | Auto Analyzer II (Technicon Instruments Corp | No information | No information |
| Mackey et. al. (2015) (sdLDL) | Plasma | Unspecified | NMR |  | NMR has the advantage of being able to determine the size and number of LDL particles, but it is very costly. |
| Mykkanen et. al. (1999) (sdLDL) | Serum | -70 | 2-14% polyacrylamide gel |  | Limitations include expensive equipment, complicated laboratory techniques, and long assay times. |
| Otvos et. al. (2006) (sdLDL) | Plasma | -80 | NMR |  | NMR has the advantage of being able to determine the size and number of LDL particles, but it is very costly. |
| Russo et. al. (2014) (sdLDL) | Plasma | -80 | Hitachi 911 automatic analyzer provided by Denka Seiken | Automated homogenous assay developed by Hirano et al. that dissociates non-LDL lipoproteins and protects sdLDL particles. |  |
| Siddiqui et. al. (2019) (sdLDL-C) | Unspecified | Unspecified | NMR |  | NMR has the advantage of being able to determine the size and number of LDL particles, but it is very costly. |
| Tsai et. al. (2014) (sdLDL-C) | Serum | -70 | Automated homogenous assay provided by Denka Seiken | Homogenous assay developed by Ito et al. that dissociates non-LDL lipoproteins and protects sd-LDL particles. Cholesterol concentration is then measured by a standard assay. | This method has good agreement with the ultracentrifugation methods, a standard procedure for the measurement of sdLDL. There was some evidence of compositional changes of sdLDL particles (d < 1.044 kg/L) using this method. |
| Williams et. al. (2013) (sdLDL) | Plasma | Unspecified | 2-14% polyacrylamide gel |  | Limitations include expensive equipment, complicated laboratory techniques, and long assay times. |
| Xu et. al. (2015) (sdLDL/sdLDL-C) | Plasma | -80 | 3% polyacrylamide gel and Lipoprint HDL system | Polyacrylamide gel electrophoresis-based method to measure cholesterol in lipoprotein fractions | Limitations include expensive equipment, complicated laboratory techniques, and long assay times. |
| Zeljkovic et. al. (2008) (sdLDL) | Plasma | -80 | 3-31% polyacrylamide gel |  | Limitations include expensive equipment, complicated laboratory techniques, and long assay times. |

**S6 Table.** Summary of univariate meta-regressions adjusted for study design of sdLDL studies

| **Covariate (n)** | **Risk of CHD (β)** | **95% CI** | **p-value** | **R^2^** |
| --- | --- | --- | --- | --- |
| Location |  |  |  | 0% |
| East Asia (Reference) (4) | 0.54 | (0.31, 0.77) | <0.01* |  |
| Europe (5) | -0.45 | (-0.74, -0.17) | 0.0016* |  |
| North America (5) | -0.06 | (-0.39, 0.27) | 0.73 |  |
| Assay Method^†^ |  |  |  | 0% |
| NMR (Reference) (3) | 0.46 | (0.18, 0.74) | 0.0009* |  |
| Analyzer (5) | -0.11 | (-0.46, 0.24) | 0.529 |  |
| Electrophoresis (6) | -0.22 | (-0.57, 0.13) | 0.214 |  |
| Population Type^†^ |  |  |  | 12.7% |
| Healthy (Reference) (2) | 0.44 | (0.14, 0.75) | 0.0033* |  |
| Unhealthy (12) | -0.18 | (-0.50, 0.14) | 0.27 |  |
| Publication Year | -0.06 | (-0.09, -0.04) | <0.01* | 15.7% |

*Significant at p<0.05 ^†^Assay methods include NMR = nuclear magnetic resonance imaging, Analyzer = automated chemical analyzer; Electrophoresis = gel electrophoresis; R^2^ = Percentage of additional heterogeneity accounted for by covariate after adjusting for study design

**S7 Table.** Summary of univariate meta-regressions adjusted for study design of sdLDL-C studies

| **Covariate (n)** | **Risk of CHD (β)** | **95% CI** | **p-value** | **R^2^** |
| --- | --- | --- | --- | --- |
| Location |  |  |  | 55.8% |
| East Asia (Reference) (4) | 0.016 | (-0.03, 0.06) | 0.0708 |  |
| Europe (1) | 0.32 | (0.07, 0.56) | 0.01 |  |
| North America (3) | 0.48 | (0.26, 0.69) | <0.01* |  |
| Assay Method^†^ |  |  |  | 0% |
| NMR (Reference) (1) | 2.44 | (0.34, 4.55) | 0.022* |  |
| Analyzer (5) | -1.84 | (-3.98, 0.29) | 0.09 |  |
| Electrophoresis (2) | -2.28 | (-4.44, -0.12) | 0.04* |  |
| Population Type^†^ |  |  |  | 72% |
| Healthy (Reference) (5) | 0.47 | (0.31, 0.62) | <0.01* |  |
| Unhealthy (3) | -0.45 | (-0.62, -0.29) | <0.01* |  |
| Publication Year | 0.077 | (-0.002, 0.16) | 0.054 | 0% |

*Significant at p<0.05; ^†^Unhealthy population consists of either samples of hospital, diabetes, HIV, liver transplant recipient patients, veterans, or the elderly; R^2^ = Percentage of additional heterogeneity accounted for by covariate after adjusting for study design

**S8 Table.** Summary of limitations of included studies that may result in risk of bias

| **Study** | **Study Design (n)** | **Measure of Association (95% CI)** | **Key Limitations** |
| --- | --- | --- | --- |
| Arai et. al. (2013) | PC (2034) | 1.54 (0.30, 7.83) | Only one measurement of sdLDL-C performed; Reasons for missing data not provided |
| Arsenault et. al. (2007) | NCC (2955) | 1.33 (1.09, 1.63)* | Nothing severe |
| Bucher et. al. (2012) | NCC (490) | 1.04 (0.99, 1.1) | Small sample size; Controls selected from at-risk set with no documented CHD (but may still have had high sdLDL-C biasing estimate towards null) |
| Goliasch et. al. (2011) | CC (302) | 1.06 (.84, 1.33) | Small sample size; Patient population <40yr, so there is potential for CAD to manifest differently; Controls were recruited from hospital so may have had other comorbidities; Did not adjust for other lipids |
| Higashioka et. al. (2019) | PC (3080) | 5.41 (2.12, 13.82)* | Only one measurement of sdLDL-C performed; Other risk factors may have changed during long follow-up; Did not adjust for other lipids |
| Hoogeveen et. al. (2015) | PC (10225) | 1.68 (1.42, 1.99)* | Reasons for missing data not provided |
| Koba et. al. (2002) Atherosclerosis | CC (348) | 5 (2.9, 8.4)* | Did not provide adjusted analysis; Did not account for statin-drug usage |
| Koba et. al. (2002) Am Heart J | CC (874) | 2.9 (1.9, 4.5)* | Did not provide adjusted analysis; Did not account for statin-drug usage |
| Koba et. al. (2008) | CC (871) | 1.027 (1.003, 1.051)* | Inefficient study design with less controls than cases |
| Kuller et. al. (2002) | CC (373) | 1.77 (1.12, 2.30)* | Did not report adjusted measure of association; Did not justify why they defined small LDL at a diameter of 18 mm when the standard is 25 mm. |
| Kwon et. al. (2006) | CC (504) | 2.312 (1.512, 3.537)* | Did not provide adjusted analysis; Inefficient study design with less controls than cases |
| Lamarche et. al. (1997) | NCC (2103) | 1.35 (0.97, 1.89) | Did not adjust for other lipids |
| Mackey et. al. (2015) | NCC (677) | 1.04 (0.77, 1.39) | Did not adjust for other lipids |
| Mykkanen et. al. (1999) | NCC (258) | 0.96 (0.49, 1.89) | Small sample size; Did not provide adjusted analysis; Studied elderly patients; Low number of cases due to short follow up; Follow up methodology unspecified; Used relative risk instead of odds ratio |
| Otvos et. al. (2006) | RCT (1061) | 1.11 (0.98, 1.27) | Did not adjust for other lipids |
| Russo et. al. (2014) | PC (95) | 1.224 (1.012, 1.492)* | Small sample size; Did not adjust for other lipids or other traditional risk factors; Selection of controls and cases unspecified |
| Siddiqui et. al. (2019) | PC (130) | 4.657 (1.335, 16.239)* | Small sample size; Low number of cases due to short follow up; Did not account for lipid-lowering therapy |
| Tsai et. al. (2014) | PC (3334) | 2.42 (1.32, 4.44)* | Nothing severe |
| Williams et. al. (2013) | RCT (142) | 1.25 (0.7, 2.24) | Relatively short follow up of a year; small sample size |
| Xu et. al. (2015) | CC (413) | 1.07 (0.99, 1.14) | Did not adjust for lipids |
| Zeljkovic et. al. (2008) | CC (359) | 2.867 (1.516, 5.425)* | Did not adjust for lifestyle risk factors |

Maximally adjusted measures of association

* Significant at p < 0.05
